# Latent multinomial models for extended batch‐mark data

**DOI:** 10.1111/biom.13789

**Published:** 2022-11-22

**Authors:** Wei Zhang, Simon J. Bonner, Rachel S. McCrea

**Affiliations:** ^1^ School of Mathematics and Statistics University of Glasgow Glasgow UK; ^2^ Department of Statistical and Actuarial Sciences University of Western Ontario London ON Canada; ^3^ Department of Mathematics and Statistics Lancaster University Lancaster UK

**Keywords:** batch marking, capture–recapture, golden mantella, latent multinomial model, saddlepoint approximation

## Abstract

Batch marking is common and useful for many capture–recapture studies where individual marks cannot be applied due to various constraints such as timing, cost, or marking difficulty. When batch marks are used, observed data are not individual capture histories but a set of counts including the numbers of individuals first marked, marked individuals that are recaptured, and individuals captured but released without being marked (applicable to some studies) on each capture occasion. Fitting traditional capture–recapture models to such data requires one to identify all possible sets of capture–recapture histories that may lead to the observed data, which is computationally infeasible even for a small number of capture occasions. In this paper, we propose a latent multinomial model to deal with such data, where the observed vector of counts is a non‐invertible linear transformation of a latent vector that follows a multinomial distribution depending on model parameters. The latent multinomial model can be fitted efficiently through a saddlepoint approximation based maximum likelihood approach. The model framework is very flexible and can be applied to data collected with different study designs. Simulation studies indicate that reliable estimation results are obtained for all parameters of the proposed model. We apply the model to analysis of golden mantella data collected using batch marks in Central Madagascar.

## 1 INTRODUCTION

Standard models for capture–recapture data, like the closed‐population models of Otis et al. (1978) and the Cormack–Jolly–Seber model (Cormack, 1964; Jolly, 1965; Seber, 1965), rely on the fact that marked individuals can be uniquely identified when they are recaptured. However, there are many experiments in which this is not possible either because it is too costly or too difficult to apply individual marks. Examples include fisheries research in which many thousands of smolts (young fish) may be captured and marked at the same time or the study of mosquitoes and other insects which are too small to mark individually (see, e.g., Davidson et al., 2019; Doll et al., 2021). In these cases, it is common to apply batch marks such that all individuals captured on one or more occasions receive identical marks. This strategy provides complete information in the case of a two‐stage experiment in which individuals are captured and marked on one occasion and recaptured on the second occasion. The standard estimators for such data, the Lincoln–Petersen and Chapman estimators, do not rely on individual identification. However, information is lost if the study comprises more than two occasions because the capture history of individuals cannot be determined uniquely. This is referred to as an extended batch‐mark study (Huggins et al. 2010; Cowen et al. 2017).

This paper was motivated by the analysis of data from a batch marking study of golden mantella (*Mantella aurantiaca*), configured as a robust design (Pollock, 1982) including six primary periods each containing 3–4 secondary occasions (21 secondary occasions in total). The golden mantella is a critically endangered frog found only in small areas of forest in Central Madagascar. Information on population status is urgently needed to inform conservation measures, but the small size of the frog makes individual marking difficult. However, batch marking using Visible Implanted Elastomers (VIE tags) is possible and was used to mark batches of frogs at 2‐month intervals during the rainy season, with a view to estimating abundance.

Modeling data from extended batch‐mark experiments is challenging because the actual capture histories for marked individuals required by common capture–recapture models cannot be observed. Observed data for such experiments comprise a set of counts including the numbers of individuals first marked, marked individuals that are recaptured, and unmarked individuals captured but released without being marked (applicable to some studies) on each capture occasion. An immediate solution is to identify all possible sets of the true (latent) individual capture histories that could have produced the observed data and then calculate the likelihood by summing up the probabilities for each set of latent capture histories. However, if the study contains more than a few capture occasions and the number of individuals marked is not very small, then there will be many configurations of the possible latent capture histories and computing the likelihood directly will be computationally expensive and thus infeasible in practice.

Huggins et al. (2010) proposed a pseudo‐likelihood approach for modeling batch mark data of marked individuals in the context of open populations. Survival and capture probabilities are estimated using estimating equations and population size is estimated through the Horvitz–Thompson estimator. Cowen et al. (2014) formulated a likelihood function for data from marked individuals and showed that their approach produces more accurate estimates and lower standard errors than the pseudo‐likelihood approach of Huggins et al. (2010). The latter is also more advantageous in terms of efficiency for larger problems (e.g., more than 11 capture occasions). These methods focus on marked individuals only; individuals captured with no marks are not included in the analysis. This gap was later filled by Cowen et al. (2017) who developed a flexible hidden Markov model (HMM) framework that accounts for data from both marked and unmarked individuals. Key to constructing the HMM for batch mark data is defining two sets of latent variables: the numbers of individuals with different batch marks that are available for capture on each occasion, and the numbers of unmarked individuals that are present in the population on each occasion. One appealing advantage of the HMM approach is that the likelihood can be maximized efficiently using the forward algorithm for HMMs.

Although the HMM approach of Cowen et al. (2017) has advantages over previous methods, we foresee some potential practical issues adapting it for our mantella data analysis. As noted by the authors (Cowen et al., 2017, Section 7.2, p. 1328), the HMM approach will encounter dimensionality issues when the numbers of marked and/or unmarked individuals become large. This occurs because a large number of marked/unmarked individuals results in high‐dimensional state‐dependent probability and transition probability matrices for the HMMs. The weather loach example considered by Cowen et al. (2017) consists of 11 occasions with at most 280 marked individuals and the largest estimated abundance of 1007 on a single occasion. As a comparison, our data consist of 21 occasions with 1090 individuals marked in the first period, and results from our model (see details in Section 5) show that the lowest abundance estimate is 1385 for a single period. Thus, we anticipate that the dimensionality issue will be much more severe if we adapt the HMM approach for our data. Cowen et al. (2017) handled the dimensionality issue in a trial‐and‐error manner by grouping the latent states into bins and putting an upper bound for the number of unmarked individuals in the population. These were proven to be useful for their example, but it is challenging in practice to determine appropriate values for the bin size and the upper bound for the number of unmarked individuals.

We propose a new model to analyze extended batch‐mark data, which avoids the practical issues of the HMM approach. The model falls within the class of latent multinomial models (Link et al., 2010), where the observed vector of counts is assumed to arise from a non‐invertible linear transformation of a latent vector that is modeled via a multinomial distribution. More specifically, we can model the true but unobservable capture–recapture process using a multinomial model, and then link the latent vector of frequencies of capture–recapture histories to the observed counts through a derived known matrix. There are two main reasons to develop the model here. First, the model framework is very flexible and can be easily adapted to analysis of different types of extended batch‐mark data. Second, the model can be fitted via an efficient maximum likelihood approach based on the saddlepoint approximation (Zhang et al., 2019, 2021).

## 2 DATA

The data on the golden mantella were collected during their breeding seasons, December through March, in the austral summers of 2014–2015 and 2015–2016. Individuals were captured during three primary periods in each year, one each in December, January, and March, with each primary period comprising three secondary occasions in the first year and four secondary occasions in the second. A total of 2,730 individuals were marked, with 1,500 marked in the first year and 1,230 in the second. The number of unmarked individuals captured on each secondary occasion ranged from a minimum of 21 on the fourth secondary occasion of the final primary period to a maximum of 438 on the second secondary occasion of the first primary period. The total number of recaptures of marked individuals was 1326. The highest number of recaptures, 651, came from individuals marked during the first primary period, which is not surprising as these individuals have the most opportunities to be recaptured. Only one individual marked during the final primary period was recaptured on one of the subsequent secondary occasions. Table 1 provides a summary of the data on marking and recaptures by the primary period.

**TABLE 1:** Data summary

**Period**	**Marks**	**1**	**2**	**3**	**4**	**5**	**6**
1	1090	219	55	17	255	90	15
2	295		43	42	41	62	37
3	115			35	7	2	0
4	686				174	81	30
5	403					107	13
6	141						1

*Notes*: Summary of the golden mantella data. The Marks column indicates how many individuals were marked over all occasions within each primary period. The columns to the right show how many times these individuals were recaptured on the subsequent secondary occasions within that same period and in each of the following periods.

## 3 MODELS AND METHODS

### 3.1 Latent process

The latent (unobservable) process for the capture–recapture study of interest using batch marks can be described as a POPAN model (Schwarz & Arnason, 1996) incorporating the robust design (Pollock, 1982). Suppose the study consists of *K* primary periods indexed by k=1,…,K, and within period *k* there are $T_{k}$ secondary capture occasions indexed by $t=1,\text{…},T_{k}$. The model assumes that the population is closed within each primary period but allows for immigration/birth and emigration/death between two primary periods. As is standard for Jolly–Seber based models, immigration/birth is assumed to be completed at the beginning of each primary period, and emigration is assumed to be permanent.

Let $\omega_{ikt}$ denote the latent (true) capture event for individual i=1,…,N on occasion *t* of period *k*, where *N* represents the size of the superpopulation that consists of all individuals which are ever present in the population and are available for capture. There are two possibilities for each $\omega_{ikt}$: 0 (non‐capture) and 1 (capture). Let $\omega_{ik}=\left(\omega_{ik1},\text{…},\omega_{ikT_{k}}\right)$ denote the latent capture history for individual *i* in primary period *k*, and $\omega_{i}=\left(\omega_{i1},\text{…},\omega_{iK}\right)$ the overall latent capture history for the individual. Then, each latent capture history ω is a vector of length $T=\sum_{k=1}^{K}T_{k}$. The number of all latent histories is $J=2^{T}$. For convenience, we index these latent histories as history j=1,…,J.

Suppose $x_{j}$ is the number of individuals with latent capture history *j*. Let $\pi_{j}=\pi_{j}\left(\theta \right)$ denote the probability that an individual has latent history *j*, where θ is a vector of model parameters. Assuming independence between individuals yields a multinomial model for $x={\left(x_{1},\text{…},x_{J}\right)}^{\prime }$, x∼Multinomial(N;π), where $\pi ={\left(\pi_{1},\text{…},\pi_{J}\right)}^{\prime }$.

Now, we consider how to express each element $\pi_{j}$ of π in terms of the model parameters θ, which include

- $p_{kt}$: the capture probability on secondary occasion *t* of period *k*; $p=(p_{11},p_{12},\text{…},p_{KT_{K}});$
- $\phi_{k}$: the survival probability from period *k* to k+1; $\phi =(\phi_{1},\text{…},\phi_{K-1});$
- $\beta_{k}$: the probability of entry in period *k*; $\beta =(\beta_{1},\text{…},\beta_{K})$.

The probabilities of events 0 and 1 on secondary occasion *t* of period *k* are $1-p_{kt}$ and $p_{kt}$, conditional on the individual being available for capture. The parameter $\phi_{k}$ denotes the probability that an individual is alive (i.e., available for capture) during period k+1 given that it was available in period *k*, and $\beta_{k}$ denotes the probability that an individual is first available for capture during period *k*. Given that emigration is permanent, β_1_ is the probability that an individual is available for capture during the first primary period, β_2_ is the probability that an individual is available for capture during the second primary period given that it was not available during the first primary period, etc. The capture event 0 has a probability of 1 on any occasion on which an individual is not available for capture, either because it has not entered or has already died/emigrated. Consider a simple example with K=3 and $T_{k}=2$ for k=1,2,3. The probability of latent history 001010 is $\text{Pr}\left(001010\right)=\left\{\beta_{1}\left(1-p_{11}\right)\left(1-p_{12}\right)\phi_{1}+\beta_{2}\right\}p_{21}\left(1-p_{22}\right)\phi_{2}p_{31}\left(1-p_{32}\right)$.

Note that the survival and capture probabilities are actually modeled on the logit scale to avoid the problem of constrained optimization when fitting the resulting model via maximum likelihood (introduced below). We also transform the entry probabilities, $\beta_{k},k=1,\text{…},K$, but more consideration is needed because of the added constraint that $\sum_{k=1}^{K}\beta_{k}=1$. Specifically, we reparameterize the model in terms of the conditional entry probabilities, $\beta_{1}^{*},\text{…},\beta_{K-1}^{*}$ defined such that $\beta_{1}^{*}=\beta_{1}$, $\beta_{2}^{*}=\beta_{2}/\left(1-\beta_{1}\right)$, …, $\beta_{K-1}^{*}=\beta_{K-1}/\left(1-\beta_{1}-\cdots -\beta_{K-2}\right)$. Optimization is then conducted with respect to $\text{logit}\left(\beta_{1}^{*}\right),\text{…},\text{logit}\left(\beta_{K-1}^{*}\right)$ which automatically constrains the value of $\beta_{K}$ so that $\sum_{k=1}^{K}\beta_{k}=1$ and $\beta_{k}\in \left(0,1\right)$ for all k=1,…,K.

### 3.2 Observed data

When batch marks are used for the study, the vector x cannot be observed because marked individuals are not identifiable. Instead, we can only observe the set of counts including:

- $m_{kt}$, the number of individuals marked on secondary occasion *t* of primary period *k*;
- $n_{kjt}$, the number of individuals that are marked in primary period *k* and recaptured on secondary occasion *t* of primary period j;

for each k=1,…,K, j=1,…,K, and $t=1,\text{…},T_{k}$. Let $m={\left(m_{11},\text{…},m_{1T_{1}},\text{…},m_{KT_{K}}\right)}^{\prime }$ and $n={\left(n_{111},\text{…},n_{KKT_{K}}\right)}^{\prime }$. Note that some elements of n are always equal to zero, specifically $n_{kjt}=0$ if j<k or both j=k and t=1. These elements are removed from n and are not regarded as data.

### 3.3 Connecting the observed and latent variables

Let $h_{1}\left(\omega \right)$ and $h_{2}\left(\omega \right)$ denote the primary period and secondary occasion within this primary period, respectively, on which an individual with true capture history ω is first captured (and marked). Let $h\left(\omega \right)=(h_{1}\left(\omega \right),h_{2}\left(\omega \right))$. It is noted that $m_{kt}=\sum_{i=1}^{N}I\left\{h\left(\omega_{i}\right)=\left(k,t\right)\right\}=\sum_{\omega \in \Omega }x_{\omega }I\left\{h\left(\omega \right)=\left(k,t\right)\right\}$, where $x_{\omega }$ denotes the number of individuals with true capture history ω, Ω is the set of all latent capture histories, and I(·) is the usual indicator function. This means that each element of m can be written as a linear transformation of the latent vector x and so we can define

(1) m=Ax,

where A is a known matrix with only 0 and 1 entries. Similarly, a linear relationship between n and x can be derived. If k<j, then $n_{kjt}=\sum_{\omega \in \Omega }x_{\omega }I\left\{h_{1}\left(\omega \right)=k\right\}I\left(\omega_{jt}=1\right)$. If k=j, then $n_{kjt}=\sum_{\omega \in \Omega }x_{\omega }I\left\{h_{1}\left(\omega \right)=k,h_{2}\left(\omega \right)<t\right\}I\left(\omega_{jt}=1\right)$. It follows that we can construct a known matrix B such that

(2) n=Bx.

Combining Equations (1) and (2) gives y=Tx where $y={\left(m^{\prime },n^{\prime }\right)}^{\prime }$ denotes the concatenated vector of the observed counts and $T={\left(A^{\prime },B^{\prime }\right)}^{\prime }$ is the matrix formed by stacking A and B. Since x follows a multinomial distribution and T is a known matrix, the model falls within the class of latent multinomial models (Link et al., 2010).

### 3.4 Unmarked individuals

The framework presented above does not consider the case in which some individuals are captured but are released without being marked due to time, cost or other constraints (Cowen et al., 2017), because this does not exist in the golden mantella data that motivated this study. However, unmarked individuals can be readily incorporated into the modeling framework here. We describe this in more detail in Section A of the Supporting information.

### 3.5 Inference

We compute the maximum likelihood estimates and standard errors for the parameters based on the saddlepoint approximation to the probability mass function of Y, the random variable associated with the observed vector y. This approach has been applied previously to latent multinomial models allowing for identification errors by Zhang et al. (2019, 2021). Briefly, if the moment generating function of X is $M_{X}\left(r\right)$, which can be computed explicitly for the multinomial distribution, then the moment generating function of Y=TX can be computed as $M_{Y}\left(s\right)=M_{X}\left(T^{\prime }s\right)$. The saddlepoint approximation to the likelihood function, first introduced by Daniels (1954), is ${\tilde{f}}_{Y}\left(y;\theta \right)=\frac{1}{{\left(2\pi \right)}^{L/2}{\left|K_{Y}^{\prime \prime }\left(\hat{s};\theta \right)\right|}^{1/2}}\exp\left\{K_{Y}\left(\hat{s};\theta \right)-{\hat{s}}^{\prime }y\right\}$ where θ denotes the vector of all parameters (as above), $K_{Y}\left(s;\theta \right)=\log\left\{M_{Y}\left(s;\theta \right)\right\}$ denotes the cumulant generating function of Y, $|K_{Y}^{\prime \prime }\left(\hat{s};\theta \right)|$ denotes the determinant of the Hessian matrix of $K_{Y}\left(s;\theta \right)$ with respect to s and evaluated at $\hat{s}$, *L* is the length of Y, and $\hat{s}=\hat{s}\left(y,\theta \right)$ solves the saddlepoint equation

(3) $$\frac{d}{ds}K_{Y}\left(s;\theta \right)=y.$$

The approximate likelihood is then maximized to compute point estimates, and standard errors are obtained from the inverse of the Hessian matrix as in the usual normal approximation for maximum likelihood estimators.

Note that the saddlepoint equation (3) rarely has an analytic solution and is instead solved numerically by minimizing $K_{Y}\left(s;\theta \right)-s^{\prime }y$ with respect to s. In particular, we apply the method of Zhang et al. (2019) which provides the efficient computation of the saddlepoint approximation through the R package TMB (Kristensen et al., 2016). Optimization and approximation of the Hessian matrix are then conducted directly in R via the function nlminb(). To speed convergence of the optimization routine and decrease the chances of finding a local maximum, we compute initial values based on a modification of the Manly–Parr approach (Manly & Parr, 1968). Section B of the Supporting information provides details.

### 3.6 Computational issues

Two data‐related challenges arose during the modeling of mantella data using the latent multinomial approach. The first is that estimates of the survival and entry probabilities may be close to zero or one for some of the primary periods in all of the models we fit (described below). This leads to problems akin to separation in standard logistic regression models. Separation occurs when the response is completely explained by a linear combination of the covariates. In this case, the likelihood is actually divergent and continues to increase as the values of one or more of the coefficients in the linear predictor move away from 0. Optimization algorithms will end at some point returning a supposed maximum likelihood estimate, but the likelihood will in fact be non‐concave. This violates the assumptions of the standard asymptotics for maximum likelihood estimators and means that the Hessian matrix may not be invertible or, if it is, that the likelihood tends to be close to flat and the resulting standard errors produced by inverting the Hessian matrix are very large and do not accurately reflect the variance of the estimators. Often the confidence intervals (CIs) produced by the asymptotic normal approximation will cover the entire (0,1) interval, after rounding (see Agresti 2012, Section 6.5 for further details). To ensure that the likelihood is not divergent, we can penalize the likelihood by subtracting a penalty term $P=\sum_{\theta \in \Theta_{p}}\text{logit}{\left(\theta \right)}^{2}/\left(2\sigma_{p}^{2}\right)$, where $\Theta_{p}$ denotes the subset of parameters in the model that are probabilities (i.e., are constrained between 0 and 1) and $\sigma_{p}$ is a penalty tuning parameter. We set $\sigma_{p}=3$ in our simulation studies and mantella data analysis. In a Bayesian framework, we could interpret the penalties as independent priors such that $\text{logit}\left(\theta \right)∼N\left(0,\sigma_{p}^{2}\right)$ for each θ. Given $\sigma_{p}=3$, this would mean, a priori, that P(0.003<θ<0.997)≈0.95 for each $\theta \in \Theta_{p}$. This is a very small penalty but we found that it was sufficient to stop the probabilities from getting too close to 0 or 1 so that standard errors could be computed (see Sections 4 and 5). If needed, one can change the value of $\sigma_{p}$ to get a larger or smaller penalty term.

The second challenge is that larger numbers of capture occasions lead to a significant computational burden. The run times are relatively short (at least in comparison to conducting a Bayesian analysis through MCMC with data augmentation of the full population), but memory usage can be very high. Optimization of the likelihood for the most complex model of the mantella data took almost 2 h, which is not too drastic, but required 95 GB of RAM. This forced us to fit these models using a high‐performance computing cluster, which may not be available to all users. The reason why memory usage is so high is that the number of possible latent capture histories is very large. Even after removing the latent histories that could not possibly have occurred given the observed data there are still over 1.15 million latent histories that could have been realized in generating the mantella data. The result is that matrices A and B are very large and consume a lot of memory even when represented in sparse format.

As a solution, we tested the concept of prefiltering the set of latent histories by computing their probabilities based on the initial values and retaining only the 10% of histories with the highest probabilities. Results comparing the analysis of the complete and prefiltered data are provided for the application to the mantella data in Section 5. This solution is admittedly ad hoc and the results will likely depend on both the initial values and the proportion of capture histories that are retained. We discuss this further in Section 6.

## 4 SIMULATION STUDY

We ran a set of simulations to assess the performance of the proposed approach for parameter estimation. As an example, we show here the results of a simulation based on a study consisting of K=6 primary periods each with $T_{k}=2$ secondary occasions. We simulated 100 datasets with the settings of N=5,000, β=(0.10,0.24,0.11,0.12,0.18,0.25), ϕ=(0.87,0.82,0.93,0.54,0.52), and p=(0.27,0.22,0.25,0.21,0.17,0.29,0.33,0.13,0.19,0.40,0.14,0.26). We generated the values of β by simulating random numbers from a multinomial distribution with size 100 and probability 1/6 for each of six classes and then dividing the numbers by 100. ϕ and p were generated from two uniform distributions over intervals (0.5, 0.95) and (0.1, 0.4), respectively. We then fit the data‐generating model to each of the datasets using the original and penalized saddlepoint likelihoods.

Table 2 summarizes the results of the simulation study. The estimators are almost unbiased for all of the model parameters with approximately nominal CI coverage when the original saddlepoint likelihood is used for model fitting. We noted that estimates of the survival rate ϕ_3_ were often close or equal to 1, given that the true value was 0.93 in the simulation. This resulted in rather wide Wald CIs, as indicated by the high mean CI width 0.59 in the table. It is well known that the Wald approach does not work in the case of boundary estimation. Zhang et al. (2021) adopted a parametric bootstrapping method in this context for a latent multinomial capture–recapture model for misidentification, which improves the precision of inference but is more time‐consuming. Alternatively, the penalized likelihood approach is more efficient. As shown in Table 2, fitting the model using the penalized likelihood yields a negligible negative bias to the estimation of ϕ_3_ and the CI coverage rate (87%) is slightly below the nominal value. However, the mean CI width for ϕ_3_ is reduced by about 54%, which means that the precision of inference is greatly improved in the estimation results. In addition, the mean CI width for ϕ_1_ is reduced by 19% when the penalized likelihood is used, but the coverage remains at 94%. Except for ϕ_1_ and ϕ_3_, penalization does not have significant effect on the estimation results of other parameters in this simulation. In simulations where the boundary estimation issue was rare, we did not notice obvious differences between the estimation results of the original and penalized likelihoods.

**TABLE 2:** Simulation results

		**Original**				**Penalized**			
**Parameter**	**True**	**Mean**	**RMSE**	**CIC**%	**CIW**	**Mean**	**RMSE**	**CIC**%	**CIW**
*N*	5000.00	5036.75	160.01	96	614.63	5027.06	155.44	94	593.91
ϕ_1_	0.87	0.86	0.07	92	0.32	0.86	0.06	94	0.26
ϕ_2_	0.82	0.83	0.05	94	0.21	0.82	0.04	99	0.19
ϕ_3_	0.93	0.93	0.06	92	0.59	0.91	0.05	87	0.27
ϕ_4_	0.54	0.54	0.04	94	0.17	0.55	0.05	95	0.17
ϕ_5_	0.52	0.55	0.09	97	0.28	0.53	0.07	94	0.27
β_1_	0.10	0.10	0.01	98	0.06	0.10	0.01	96	0.06
β_2_	0.24	0.24	0.02	97	0.10	0.24	0.02	98	0.10
β_3_	0.11	0.11	0.02	95	0.09	0.11	0.02	94	0.09
β_4_	0.12	0.12	0.02	95	0.07	0.12	0.02	94	0.07
β_5_	0.18	0.18	0.02	93	0.06	0.18	0.02	93	0.06
β_6_	0.25	0.25	0.02	97	0.09	0.25	0.02	96	0.09
*p*_11_	0.27	0.27	0.04	99	0.17	0.28	0.04	95	0.17
*p*_12_	0.22	0.22	0.03	99	0.14	0.21	0.03	97	0.14
*p*_21_	0.25	0.25	0.02	96	0.08	0.26	0.02	94	0.08
*p*_22_	0.21	0.21	0.02	97	0.07	0.21	0.02	97	0.07
*p*_31_	0.17	0.16	0.01	95	0.05	0.17	0.01	90	0.05
*p*_32_	0.29	0.29	0.02	96	0.08	0.29	0.02	95	0.08
*p*_41_	0.33	0.33	0.02	94	0.09	0.33	0.02	95	0.08
*p*_42_	0.13	0.13	0.01	98	0.04	0.13	0.01	98	0.04
*p*_51_	0.19	0.19	0.01	93	0.06	0.19	0.01	95	0.06
*p*_52_	0.40	0.40	0.02	97	0.10	0.40	0.03	93	0.10
*p*_61_	0.14	0.13	0.02	94	0.06	0.13	0.02	94	0.06
*p*_62_	0.26	0.25	0.03	94	0.11	0.26	0.03	96	0.11

*Notes*: Parameter estimation results of a simulation study with 100 replicates in the setting of $K=6,T_{k}=2$ for k=1,…,6, N=5,000, β=(0.10,0.24,0.11,0.12,0.18,0.25), p=(0.27,0.22,0.25,0.21,0.17,0.29,0.33,0.13,0.19,0.40,0.14,0.26), and ϕ= (0.87, 0.82, 0.93, 0.54, 0.52). CIC% and CIW represent 95% confidence interval coverage, and mean 95% confidence interval width, respectively; RMSE: root mean square error.

### 4.1 Model selection

Model selection needs careful consideration when analyzing real data. However, there is not a general method available for model selection when the saddlepoint approximation is used for maximum likelihood estimation. Zhang et al. (2019) suggested that the saddlepoint‐approximation‐based Akaike information criterion (AIC) works well for model selection when the observed data of the latent multinomial models consist mostly of large counts (e.g., no less than 5), which is the case for the mantella data we analyze below. Here, we also use simulations to check the performance of AIC based on the saddlepoint likelihood for model selection under the proposed latent multinomial model for extended batch‐mark data.

We first considered the same datasets generated in the simulation study above. For each dataset, in addition to the true model, denoted by p(t)ϕ(k), we fit three simplified models denoted by p(t)ϕ(·), p(·)ϕ(k), and p(·)ϕ(·). Here, p(t) and p(·) represent the options of either completely time‐varying capture probabilities or constant capture probability over all occasions, and ϕ(k) and ϕ(·) represent the options of either period‐dependent or constant survival rates. Entry probabilities were allowed to be time‐dependent for all four models. We fit each model using both the original and penalized likelihoods, and then computed the AIC value in each case. In both cases, AIC can always correctly select the data‐generating model.

We further investigated the performance of AIC using another simulation study, where *N* was set to be 1,000, while other parameters remained the same as in the simulation above. Table 3 presents the results of model selection for this simulation. When the original saddlepoint likelihood was used for model fitting, AIC selected the data‐generating model p(t)ϕ(k) for 69 out of the 100 datasets. For the remaining 31 datasets, the simpler model p(t)ϕ(·) was favored by AIC. This indicates that AIC is conservative and able to determine the model for capture probabilities but often selects a simpler model for survival probabilities. When model p(t)ϕ(·) was preferred, the difference between the AIC values of this model and the true model was not large. The largest difference was 5.7 and 35% of the time the difference was less than 2. We observed that the AIC computed from the penalized likelihood performed similarly and selected the data‐generating model p(t)ϕ(k) for 63 of the 100 datasets, while model p(t)ϕ(·) was preferred for the remaining 37 datasets. In terms of the inability of AIC computed using the original likelihood to always determine that time‐dependent survival is necessary, we believe that this is due to a lack of power caused by batch‐marking and not collecting individual level data. The lack of power is also evident from the widths of the CIs for the survival probabilities in Table 2. The performance of AIC for model selection improves significantly for simulations with larger abundance or capture probabilities, while other parameter values remain the same as those for the simulation study here. See Tables 6 and 7 in Section C of the Supporting information.

**TABLE 3:** Model selection

**Likelihood**	p(t)ϕ(k)	p(·)ϕ(k)	p(t)ϕ(·)	p(·)ϕ(·)
Original	69	0	31	0
Penalized	63	0	37	0

*Notes*: Summary of the simulations for model selection. Each entry of the table gives the number of cases (out of 100), where the model has the lowest AIC value and is selected as the preferred model.

## 5 APPLICATION

We fit six different models to the mantella data formed by combining three alternatives for the capture probability and two for the survival probability. The three alternatives considered for the capture probability were: (1) distinct on every secondary period within each primary period (model p(t) as in Section 4.1), (2) equal for all secondary periods within each primary period (model p(k)), and (3) constant over all secondary periods (model p(·)). For the survival probability, we considered the model with a distinct parameter for each primary period (model ϕ(k) as in Section 4.1) and a model with a constant monthly survival, denoted by ϕ(m). This is a variation of the constant survival model denoted by ϕ(·) in Section 4.1 which accounts for the fact that the primary periods in the mantella study are not equally spaced. Survival between periods *k* and k+1 for this model is defined as $\phi_{k}=S^{\Delta_{k}^{m}}$, where *S* is the monthly survival rate and $\Delta_{k}^{m}$ denotes the time in months between the two periods. If the time between consecutive periods is constant, $\Delta_{k}^{m}=d$, then $\phi_{k}=s^{d}$ recovers the constant survival model, ϕ(·). No constraints were placed on the recruitment parameters in any of these models.

We also fit these models with all three of the methods described in Section 3: (1) constructing the likelihood from the complete set of latent histories without penalization (original), (2) constructing the likelihood from the complete set of latent histories with penalization (penalized), and (3) constructing the likelihood from the prefiltered set of latent histories with penalization (prefiltered). Table 4 compares the different models in terms of the fit to the data (AIC), run time, and memory usage computed with all three methods of fitting. The absolute values of the AIC are different when comparing the three variants of the same model, but the qualitative results are exactly the same. For all three methods, the AIC provides very strong support for the most complicated model, Model 2: p(t)ϕ(k). However, the model fit using the complete set of latent histories ran for almost 2 h and required almost 96 GB of RAM, while the prefiltered version ran in under 16 minutes and required less than 9 GB of RAM. This makes it feasible to fit these models on a personal computer and to reasonably compare different models to test alternative hypotheses.

**TABLE 4:** Model comparison

	**Original**			**Penalized**			**Prefiltered**		
**Model**	**AIC**	**Mem**.	**Time**	**AIC**	**Mem**.	**Time**	**AIC**	**Mem**.	**Time**
1: p(t)ϕ(m)	1131.15	95.77	96.65	1155.95	94.98	88.43	1159.92	8.33	12.73
2: p(t)ϕ(k)	1007.51	95.77	116.75	1029.34	94.94	93.43	1034.87	8.32	15.80
3: p(k)ϕ(m)	1321.31	95.47	57.25	1330.79	95.44	46.85	1334.50	9.24	11.95
4: p(k)ϕ(k)	1201.96	95.52	71.32	1212.58	95.50	59.90	1217.27	7.90	12.67
5: p(·)ϕ(m)	2645.83	95.38	39.35	2660.74	95.37	52.33	2662.63	9.25	11.08
6: p(·)ϕ(k)	1443.91	95.39	54.33	1453.14	95.33	42.27	1454.74	7.76	10.57

*Notes*: Comparisons for the six models fit to the golden mantella data retaining the complete set of latent histories without penalization (original), retaining the complete set of latent histories with penalization (penalized), or retaining only the 10% with the highest probability given the initial values with penalization (prefiltered). Each model is defined by the structure of the capture and survival probabilities. Results include the AIC, memory usage in GB, and run time in minutes.

**FIGURE 1:**
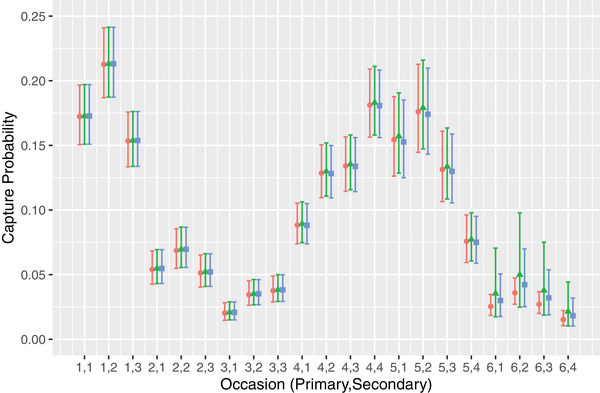
Estimated capture probabilities. Estimates of the capture probabilities from the selected model using (1) the complete set of latent histories without penalization (red circles), (2) the complete set of latent histories with penalization (green triangles), and (3) the prefiltered histories with penalization (blue squares). Vertical bars show the extents of the 95% CIs. Points for each version of the model have been offset to avoid overlap. This figure appears in color in the electronic version of this article, and color refers to that version.

Table 5 displays point estimates and CIs of the demographic parameters for the three versions of the selected model, Model 2, while Figure 1 compares the estimates of the capture probabilities. Estimates and CIs from the original fit and penalized fit were almost identical except when the estimate from the original fit lay on the boundary and the corresponding CI covered all of (0,1). In most cases, the estimate from the penalized fit was pulled slightly inside the (0,1) interval, as in the case of β_3_, and the CI narrowed to a reasonable range. The only exceptions to this are the parameters relating to the final primary period including the probability of survival from period 5 to 6 (ϕ_5_), the probability of entry in period 6 (β_6_), and the abundance during the period (*N*_6_). Penalizing the likelihood reduced the estimate of ϕ_5_ from 1 (95%CI=0,1) to 0.72 (95%CI=0.18,0.97) and of β_6_ from 0.16(95%CI=0.11,0.22) to 0.12(95%CI=0.07,0.21). These changes lead to the conclusion that there were fewer individuals alive during this period, either surviving from previous periods or entering the population in that period, and that the capture probabilities are higher. This in turn acts to reduce the estimate of abundance during this period, *N*_6_, which decreased from 2285(95%CI=1902,2746) to 1649(95%CI=855,3178), and the estimate of the super‐population size, *N*, which decreased from 5699(95%CI=5321,6133) to 5467(95%CI=5024,5995). This difference was not observed in the simulation study, and we believe that it is related to the fact that the number of recaptures during the sixth primary period was so low making the results relating to this occasion highly unstable. This may also indicate a violation of the model assumptions, which we discuss below. That said, the CIs for the abundance, both in period 6 and over all periods, overlap considerably so that there is no difference in the qualitative results.

**TABLE 5:** Point estimates

**Parameter**	**Original**	**Penalized**	**Prefiltered**
*N*	5699(5321,6133)	5467(5024,5995)	5567(5145,6063)
ϕ_1_	0.5(0.42,0.58)	0.5(0.42,0.58)	0.5(0.42,0.58)
ϕ_2_	1(0,1)	0.98(0.77,1)	0.98(0.78,1)
ϕ_3_	0.64(0.53,0.74)	0.65(0.54,0.74)	0.66(0.55,0.76)
ϕ_4_	0.36(0.29,0.44)	0.36(0.29,0.43)	0.37(0.3,0.45)
ϕ_5_	1(0,1)	0.72(0.18,0.97)	0.85(0.21,0.99)
β_1_	0.43(0.38,0.47)	0.44(0.39,0.5)	0.44(0.39,0.49)
β_2_	0.18(0.14,0.24)	0.19(0.14,0.25)	0.18(0.13,0.24)
β_3_	0(0,1)	0.01(0,0.09)	0.01(0,0.09)
β_4_	0.13(0.09,0.18)	0.13(0.09,0.18)	0.13(0.09,0.18)
β_5_	0.1(0.08,0.13)	0.11(0.09,0.14)	0.11(0.08,0.13)
β_6_	0.16(0.11,0.22)	0.12(0.07,0.21)	0.14(0.08,0.22)
*N*_1_	2431(2187,2703)	2427(2184,2696)	2427(2184,2697)
*N*_2_	2259(1915,2664)	2233(1890,2639)	2232(1891,2635)
*N*_3_	2259(1915,2664)	2227(1878,2641)	2229(1883,2639)
*N*_4_	2185(1939,2462)	2164(1922,2437)	2192(1948,2467)
*N*_5_	1385(1178,1630)	1364(1161,1602)	1403(1196,1646)
*N*_6_	2285(1902,2746)	1649(855,3178)	1948(1223,3104)

*Notes*: Point estimates and 95% confidence intervals of the demographic parameters from the selected model fit to the golden mantella data. The second and third columns provide the results from fitting with the complete set of latent histories using the original and penalized likelihoods, while the fourth column provides the results from fitting with the 10% of latent histories having the highest probabilities given the initial values.

Point estimates and CIs for all parameters from the penalized and prefiltered methods were almost identical, except again on the final period. This suggests that there was almost no loss or change in the information by removing 90% of the latent histories and that prefiltering based on the initial parameter values provides a valid approach to reduce the computational burden.

One important observation is that there seem to be patterns in the estimates that may be indicative of systematic changes that have not been accounted for by any of the proposed models. Point estimates of the recruitment probabilities show a continual decrease within each of the two breeding seasons (i.e., periods 1–3 and again in periods 4–6) and the estimated capture probabilities seem to vary in a smooth, almost seasonal fashion. We believe that this may indicate that individuals are entering and leaving the breeding grounds at different times during the breeding season, violating the assumption of closure within the primary periods. We did not explore more complicated models to account for this phenomenon in this research, and plan to do so in the future.

## 6 DISCUSSION

The latent multinomial model offers a flexible framework for modeling extended batch‐mark data. The ability to express the model in terms of the unobserved latent capture histories allows the model to accurately reflect the data‐generating process and does not require unrealistic and overly simplistic model assumptions to be made. Batch marking studies are typically more time and cost effective and can be used for species that are difficult or impossible to mark individually. We have demonstrated that it is possible to estimate key parameters of interest with good precision from this type of data.

In practice, we have observed that the model works well in both the simulated and real‐data applications. Boundary estimation issues were encountered which are overcome with appropriate penalization methods. The model is computationally efficient in terms of time, but for scenarios with large numbers of primary and secondary occasions a large amount of computer memory was required. Given that not everyone has access to high‐performance computing resources, we have demonstrated that prefiltering the possible latent capture histories to those that are most likely to occur based on initial parameter estimates reduces the required RAM.

The results of prefiltering the data will depend on both the initial parameter estimates and the proportion of latent capture histories retained. If either the initial values are far from the true value or the proportion of capture histories retained is too small then the likelihood function will be distorted too much, and the resulting inference will not be accurate. In the analysis of the mantella data, we were able to conduct the analysis with the full set of latent capture histories and confirm that the results with and without prefiltering were almost identical. However, this negates the purpose of prefiltering. If sufficient RAM is available to conduct the analysis with the full set of latent capture histories then this is always preferable. If prefiltering is performed in practice then we recommend repeating the analysis starting from multiple sets of initial parameter estimates and comparing the results. The different sets of initial parameters should be chosen so that they are diffuse within the space of possible parameters, as is the case for choosing multiple sets of initial values for standard optimization routines to reduce the chances that the algorithm reaches a local maximum/minimum. This will require that the model is fit repeatedly, but this should not represent a computational burden as the jobs could be run in parallel. If the results differ significantly then the analysis should be repeated from the same initial values but retaining a larger proportion of the latent capture histories.

As an example, we repeated fitting the selected model to the golden mantella data starting from two alternative sets of initial parameter values. These were generated by either setting $p_{1}=\cdots =p_{6}=0.10$ or $p_{1}=\cdots =p_{6}=0.40$ and then computing initial estimates for the remaining parameters as given in Section B of the Supporting information. These values were chosen as they are expected to bound the capture probabilities based on the advice of the experts in the field. Table 11 in Section D of the Supporting information presents the different sets of initial values. Table 12 and Figure 1 in Section D of the Supporting information compare the point estimates and 95% CIs of the parameters for the fitted models. The results do differ, but this is to be expected given that different sets of the latent capture histories are retained. However, the changes are small and the qualitative conclusions are practically identical. Estimates of the total population size from the new analysis are within 95 of the original estimate (a difference of <2%) and the 95% CIs overlap almost completely. Estimates of the population size by the primary period are within 110 (a difference of 5%) except for the final period when the difference is as high as 284 (nearly 15%), but these estimates are very uncertain and the 95% CI for the estimate of *N*_6_ from the original initial values is completely contained within the 95% CI computed with the initial estimate $p_{k}=0.10$, k=1,…,6. These results suggest that prefiltering is not affecting the overall conclusions of the analysis and support the results without having to fit the model including the complete set of latent capture histories.

We have observed that population size and capture probabilities are estimated well from batch‐mark data as is evident from both the simulation study and mantella application results. However, we have also seen that survival estimates are much less precise. This observation is not surprising, since estimation of survival relies on recaptures of individuals from batches of previously marked cohorts of animals and these observations will typically be fairly small relative to the number of individuals marked. The lack of individual‐level information in batch‐mark data means that the data are a lot less informative for the estimation of survival than for other types of data such as capture–recapture or ring–recovery data. We observed this through the wider CIs of survival probabilities in the simulation study. Similar results were also shown by Cowen et al. (2014) who conducted a simulation study to compare estimates from the Jolly–Seber model with complete identity information and an associated batch‐mark model in which identities were removed. They reported that estimates of the survival probabilities from batch‐mark data were between 30% and 40% as efficient as those from data with complete identities, though the exact results depended heavily on the choice of parameters. This observation should guide those planning studies to consider what the parameters of interest are when selecting which type of data they should collect.

One key advantage of the latent multinomial approach is that it is often much simpler to conceptualize the model and write the probabilities for the latent histories than the observed histories. It is clear that further adaptations could be made to the model, for example, accounting for temporary emigration from the site, which we believe would be possible due to the robust design nature of the data, following an approach similar to Zhou et al. (2019). It would also be of interest to explore how batch‐mark data could be used in conjunction with other forms of data, such as count data, to share information on common parameters and to examine the relative information contained in the different data types. Such an integrated approach may alleviate some of the high correlations observed between parameters for extended batch‐mark data alone, see, for example, Catchpole et al. (1998). The treatment of multiple data types using a latent multinomial approach may also offer a practical solution to overcome needing to assume independence between datasets.

## Supporting information

Web Appendices referenced in Sections 3.4 and 3.5, along with the code to reproduce the simulation study, are available with this paper at the Biometrics website on Wiley Online Library.
Table 1: Results of supplementary simulation 1
Table 2: Results of supplementary simulation 2
Table 3: Results of supplementary simulation 3
Table 4: Results of supplementary simulation 4
Table 5: Results of supplementary simulation 5
Table 6: Supplementary model selection 1
Table 7: Supplementary model selection 2
Table 8: Supplementary model selection 3
Table 9: Supplementary model selection 4
Table 10: Supplementary model selection 5
Table 11: Alternative initial values
Table 12: Parameter estimates 2
Figure 1: Estimated capture probabilities 3

Data S1

## Data Availability

Research data from the study of the golden mantella are not shared. Data are the property of the Madagasikara Voakajy organization and permission to release the data has not been granted. The organization can be contacted at voakajy@voakajy.mg.
