# Supplementary material for: Latent multinomial models for extended batch‐mark data
Source: Biometrics. 2022 Nov 22;79(3):2732–42. doi: 10.1111/biom.13789 (PMC10953401; doi:10.1111/biom.13789)
Supplement: Supplementary file 1 — Web Appendices referenced in Sections 3.4 and 3.5, along with the code to reproduce the simulation study, are available with this paper at the Biometrics website on Wiley Online Library. Table 1: Results of supplementary simulation 1 Table 2: Results of supplementary simulation 2 Table 3: Results of supplementary simulation 3 Table 4: Results of supplementary simulation 4 Table 5: Results of supplementary simulation 5 Table 6: Supplementary model selection 1 Table 7: Supplementary model selection 2 Table 8: Supplementary model selection 3 Table 9: Supplementary model selection 4 Table 10: Supplementary model selection 5 Table 11: Alternative initial values Table 12: Parameter estimates 2 Figure 1: Estimated capture probabilities 3 [file BIOM-79-2732-s002.pdf]

Supporting Information for
"Latent Multinomial Models for Extended Batch Mark Data"
by Wei Zhang, Simon J. Bonner, and Rachel S. McCrea

A Unmarked individuals

When unmarked individuals are considered, there are three possible events on each capture occasion:

- 0 if the individual is not captured;
- 1 if the individual is captured and marked before being released or is already marked;
- 2 if the individual is captured and released without being marked.

We now consider the set of all possible latent histories. If an individual is captured and first marked on some occasion, then the individual does not have event 1 before that occasion or event 2 after that occasion. The number of all such latent histories is $J_1 = T2^{T-1}$, where $T = \sum_{k=1}^{K} T_k$ is the total number of capture occasions. If an individual is never marked, its capture history does not contain any event 1. The number of all such latent histories is $J_2 = 2^T$. Then the total number of latent capture histories is $J = J_1 + J_2 = (T+2)2^{T-1}$. For convenience, we index these latent histories as history $j = 1, \dots, J$.

Then we consider how to express the probability for each latent capture history in terms of the model parameters $\boldsymbol{\theta}$, which include

- p_{kt} the capture probability on secondary occasion t of period k;
- ϕ_k the survival probability from period k to $k+1$;
- α the probability of not being marked before release for a captured (unmarked) individual;
- β_k the probability of entry in period k.

Using these parameters, the probabilities of events 0 and 2 on secondary occasion t of period k are $1 - p_{kt}$ and αp_{kt}. The probability of event 1 on secondary occasion t of period k is: $(1-\alpha)p_{kt}$ if the individual is first captured and marked, and p_{kt} if the individual is recaptured (previously marked). To be more general, the parameter α may also depend on the secondary occasion and/or the primary period. When specifying the formula for each π_j, we also need to consider survival between periods and the entry of the N individuals in each period. Consider a simple

example with $K = 3$ and $T_k = 2$ for $k = 1, 2, 3$. The probability of latent history 021010 is $\Pr(021010) = \beta_1(1 - p_{11})\alpha p_{12}\phi_1(1 - \alpha)p_{21}(1 - p_{22})\phi_2 p_{31}(1 - p_{32})$.

In addition to the observed counts $\boldsymbol{m}$ and $\boldsymbol{n}$ defined for the model without unmarked individuals, we also have

$$\boldsymbol{u} = (u_{11}, \ldots, u_{1T_1}, \ldots, u_{KT_K})',$$

where u_{kt} denotes the number of individuals that are captured and released without being marked on occasion t of period k. Note that

$$u_{kt} = \sum_{i=1}^{N} \mathcal{I}\left(\omega_{ikt} = 2\right) = \sum_{\boldsymbol{\omega} \in \Omega} x_{\boldsymbol{\omega}} \mathcal{I}\left(\omega_{kt} = 2\right),$$

which indicates that a matrix $\boldsymbol{C}$ can be derived such that

$$\boldsymbol{u} = \boldsymbol{C}\boldsymbol{x}.$$

It follows that we have

$$\boldsymbol{y} = \begin{pmatrix} \boldsymbol{m} \\ \boldsymbol{n} \\ \boldsymbol{u} \end{pmatrix} = \begin{pmatrix} \boldsymbol{A} \\ \boldsymbol{B} \\ \boldsymbol{C} \end{pmatrix} \boldsymbol{x} = \boldsymbol{T}\boldsymbol{x},$$

which still falls within the latent multinomial class.

B Initial Values

We compute initial values for optimizing the likelihood via a Manly-Parr type approach that first estimates the capture probabilities based only on the data within each capture period and then estimates the survival probabilities conditional on the capture probabilities. Initial values are first computed for the model assuming that the capture probabilities are constant across the secondary occasions within a primary period but allowing for variation in both the capture and survival probabilities between primary periods($p(k)$, $\phi(k)$). These values are then adjusted for any alternative models.

The key to this approach is that individual identities do not need to be known to estimate capture probabilities based solely on the data collected within each primary period. Assuming closure, we estimate the capture probability on the tth secondary occasion, $t > 1$, within the kth primary period by the proportion of individuals that were marked on the previous secondary occasions (plus 1) and recaptured on occasion t (plus 1):

$$\widetilde{p}_{kt} = \frac{n_{kkt} + 1}{\sum_{s=1}^{t-1} m_{ks} + 1}$$

Adding 1 in both the numerator and denominator avoids division by 0 if $\sum_{s=1}^{t-1} m_{ks} = 0$ and sets $\widetilde{p}_{kt} = 0.5$ in this case. We also truncated the values to the interval $(0.1, 0.9)$ to ensure that the capture probabilities are not too close to 0 or 1. Assuming that the capture probability is constant across the secondary occasions, we obtain our initial estimate of the probability of capture within primary period k by averaging these values:

$$\widetilde{p}_k = \frac{1}{T_k - 1} \sum_{t=2}^{T_k} \widetilde{p}_{kt}.$$

Our initial value for the probability of survival between primary periods k and $k+1$ is then given by

$$\widetilde{\phi}_k = \frac{n_{(k-1)k1}}{\widetilde{p}_k \sum_{t=1}^{T_{k-1}} m_{(k-1)t}}.$$

This represents the proportion of individuals marked in the $(k-1)^{th}$ primary period that are recaptured on the first secondary occasion of the next primary period, inflated by the capture probability to account for non-detections.

Initial values for models with different assumptions about the capture and survival probabilities are then obtained either by averaging or repeating these values. For example, the initial value for the survival probability in a model assuming constant survival between all primary periods is set to

$$\widetilde{\phi} = \frac{1}{K-1} \sum_{k=1}^{K-1} \widetilde{\phi}_k.$$

Alternatively, initial capture probabilities for a model allowing for variation across secondary occasions within a primary period would be set equal to $\widetilde{p}_k$ for all secondary occasions within primary period k. We do not use the values $\widetilde{p}_{kt}$ given above because these can be very imprecise, especially for secondary occasions early in the primary period when the number of previously marked individuals may be small.

Initial estimates of the abundance in each primary period are then computed from Horvitz-Thompson type estimates of the abundance on each secondary occasion. Let $c_{kt} = m_{kt} + u_{kt} + \sum_{l=1}^{k} n_{lkt}$ denote the total number of individuals captured on the tth secondary occasion within the kth primary period. The Horvitz-Thompson estimate of abundance on this occasion would then be

$$\widetilde{N}_{kt} = \frac{c_{kt}}{\widetilde{p}_k}.$$

Assuming closure there can only be one estimate of abundance within the primary period and so we set

$$\widetilde{N}_k = \text{Median}(\widetilde{N}_{k1}, \ldots, \widetilde{N}_{kT_k}).$$

Here we consider the median instead of the mean because the distribution of the Horvitz-Thompson estimator is generally right-skewed and can produce very large estimates of abundance when the capture probability is small. Finally, we compute initial values for the number of individuals entering on each primary period by estimating the number of individuals surviving from the previous period and subtracting this from the estimated abundance. That is, we set $\widetilde{B}_1 = \widetilde{N}_1$ and

$$\widetilde{B}_k = \widetilde{N}_k - \widetilde{\phi}_k \widetilde{N}_{k-1}$$

for $k = 2, \ldots, K$. The initial value for the probability of entry in primary period k is then

$$\widetilde{\beta}_k = \frac{\widetilde{B}_k}{\sum_{l=1}^{K} \widetilde{B}_k}$$

for $k = 1, \ldots, K$.

C Further Simulation Results

Tables 1–5 present more simulation results in different settings from those in the main text. Similarly, reliable estimation results are obtained for all model parameters in all these simulations. Note that using the penalized likelihood helps to reduce the mean confidence interval (CI) widths for those survival parameters with true values being over 0.8 in the simulations. For other parameters, using penalization only has negligible effects.

Tables 6–10 present more simulation results on model selection using AIC. Compared to the results shown in Table 3 in the main text, the performance of AIC is significantly improved if either capture probabilities or abundance N increases in the simulations while other parameters remain unchanged; see Tables 6 and 7. This is because in these cases we have more captures of individuals and thus the estimation of survival rates becomes more accurate, which helps to select the right model for survival rates.

Table 10 presents results in an extreme case, where capture probabilities are in an increasing order while survival rates are decreasing as time goes. This makes it more difficult to estimate the capture and survival probabilities accurately, which is also evident from the mean widths of the CIs for the survival probabilities in Table 5. From Table 10, we can see that AIC is conservative and able to determine the model for capture probabilities but often selects a simpler model for the survival probabilities. Similar to the case shown in the main text, we believe that this is due to a lack of power caused by batch-marking and not collecting individual level data.

Table 1: Results of Supplementary Simulation 1

		Original				Penalized			
	True	Mean	RMSE	CIC%	CIW	Mean	RMSE	CIC%	CIW
N	1000.00	1004.21	51.33	97	207.62	995.99	45.41	97	192.48
ϕ_1	0.87	0.88	0.10	95	0.68	0.85	0.09	94	0.46
ϕ_2	0.82	0.83	0.09	94	0.46	0.83	0.07	94	0.35
ϕ_3	0.93	0.91	0.09	90	0.76	0.88	0.08	86	0.43
ϕ_4	0.54	0.55	0.09	94	0.32	0.56	0.09	96	0.31
ϕ_5	0.52	0.54	0.14	98	0.47	0.53	0.12	96	0.43
β_1	0.10	0.10	0.03	96	0.12	0.10	0.03	96	0.11
β_2	0.24	0.24	0.05	98	0.18	0.24	0.04	98	0.17
β_3	0.11	0.11	0.04	94	0.19	0.11	0.04	92	0.16
β_4	0.12	0.12	0.03	91	0.13	0.12	0.03	92	0.12
β_5	0.18	0.18	0.03	93	0.12	0.18	0.03	93	0.11
β_6	0.25	0.25	0.04	95	0.16	0.24	0.04	96	0.15
p_{11}	0.32	0.32	0.08	100	0.35	0.33	0.08	99	0.34
p_{12}	0.27	0.26	0.06	99	0.30	0.27	0.06	99	0.30
p_{21}	0.30	0.31	0.05	93	0.17	0.31	0.05	93	0.17
p_{22}	0.26	0.26	0.04	92	0.15	0.27	0.04	91	0.15
p_{31}	0.22	0.21	0.03	95	0.12	0.22	0.03	95	0.12
p_{32}	0.34	0.34	0.04	97	0.16	0.35	0.04	98	0.16
p_{41}	0.38	0.38	0.04	95	0.17	0.39	0.04	97	0.17
p_{42}	0.18	0.18	0.03	96	0.10	0.19	0.03	93	0.10
p_{51}	0.24	0.24	0.04	94	0.13	0.24	0.04	94	0.13
p_{52}	0.45	0.45	0.06	95	0.21	0.45	0.06	96	0.21
p_{61}	0.19	0.19	0.04	97	0.15	0.19	0.04	97	0.15
p_{62}	0.31	0.31	0.06	97	0.23	0.32	0.05	97	0.23

Parameter estimation results of a simulation study with 100 replicates in the setting of $K = 6, T_k = 2$ for $k = 1, \ldots, 6$, $N = 1000$, $\boldsymbol{\beta} = (0.10, 0.24, 0.11, 0.12, 0.18, 0.25)$, $\boldsymbol{\phi} = (0.87, 0.82, 0.93, 0.54, 0.52)$, and $\boldsymbol{p} = (0.32, 0.27, 0.30, 0.26, 0.22, 0.34, 0.38, 0.18, 0.24, 0.45, 0.19, 0.31)$. RMSE: root mean square error. CIC%, and CIW represent 95% confidence interval coverage, and mean 95% confidence interval width.

Table 2: Results of Supplementary Simulation 2

		Original				Penalized			
	True	Mean	RMSE	CIC%	CIW	Mean	RMSE	CIC%	CIW
N	2000.00	2036.42	104.55	99	418.34	2019.11	88.84	100	384.91
ϕ_1	0.87	0.87	0.10	91	0.59	0.85	0.09	92	0.39
ϕ_2	0.82	0.84	0.08	95	0.36	0.84	0.07	98	0.30
ϕ_3	0.93	0.93	0.08	91	0.75	0.90	0.07	88	0.39
ϕ_4	0.54	0.54	0.08	94	0.26	0.55	0.07	93	0.26
ϕ_5	0.52	0.57	0.13	98	0.45	0.55	0.11	97	0.41
β_1	0.10	0.10	0.03	95	0.11	0.10	0.03	94	0.10
β_2	0.24	0.24	0.05	95	0.17	0.24	0.04	96	0.15
β_3	0.11	0.11	0.03	96	0.17	0.11	0.03	95	0.14
β_4	0.12	0.12	0.03	99	0.12	0.12	0.03	99	0.11
β_5	0.18	0.17	0.02	96	0.10	0.18	0.02	96	0.10
β_6	0.25	0.26	0.04	96	0.15	0.26	0.03	97	0.14
p_{11}	0.27	0.27	0.07	98	0.26	0.27	0.07	98	0.26
p_{12}	0.22	0.22	0.06	96	0.22	0.23	0.06	96	0.22
p_{21}	0.25	0.26	0.03	98	0.12	0.26	0.03	97	0.12
p_{22}	0.21	0.21	0.03	92	0.11	0.22	0.03	93	0.11
p_{31}	0.17	0.16	0.02	93	0.08	0.16	0.02	93	0.08
p_{32}	0.29	0.29	0.03	96	0.12	0.29	0.03	97	0.11
p_{41}	0.33	0.33	0.03	95	0.13	0.34	0.03	97	0.13
p_{42}	0.13	0.13	0.02	95	0.06	0.13	0.02	96	0.06
p_{51}	0.19	0.20	0.02	96	0.09	0.20	0.02	96	0.09
p_{52}	0.40	0.40	0.04	91	0.16	0.40	0.04	91	0.16
p_{61}	0.14	0.13	0.03	95	0.10	0.14	0.02	97	0.10
p_{62}	0.26	0.25	0.04	97	0.18	0.26	0.04	97	0.17

Parameter estimation results of a simulation study with 100 replicates in the setting of $K = 6, T_k = 2$ for $k = 1, \ldots, 6$, $N = 2000$, $\boldsymbol{\beta} = (0.10, 0.24, 0.11, 0.12, 0.18, 0.25)$, $\boldsymbol{\phi} = (0.87, 0.82, 0.93, 0.54, 0.52)$, and $\boldsymbol{p} = (0.27, 0.22, 0.25, 0.21, 0.17, 0.29, 0.33, 0.13, 0.19, 0.40, 0.14, 0.26)$. RMSE: root mean square error. CIC%, and CIW represent 95% confidence interval coverage, and mean 95% confidence interval width.

Table 3: Results of Supplementary Simulation 3

		Original				Penalized			
	True	Mean	RMSE	CIC%	CIW	Mean	RMSE	CIC%	CIW
N	2000.00	2000.04	32.05	94	127.16	1998.91	31.94	94	126.67
ϕ_1	0.76	0.77	0.06	97	0.24	0.77	0.06	97	0.23
ϕ_2	0.50	0.50	0.04	94	0.17	0.50	0.04	95	0.17
ϕ_3	0.63	0.64	0.05	95	0.20	0.64	0.05	95	0.19
ϕ_4	0.62	0.63	0.06	94	0.21	0.63	0.06	94	0.21
β_1	0.23	0.23	0.02	93	0.07	0.23	0.02	94	0.07
β_2	0.21	0.21	0.02	93	0.08	0.21	0.02	93	0.07
β_3	0.28	0.28	0.02	95	0.09	0.28	0.02	95	0.09
β_4	0.14	0.14	0.02	96	0.08	0.14	0.02	96	0.08
β_5	0.14	0.14	0.01	94	0.05	0.14	0.01	94	0.05
p_{11}	0.34	0.35	0.03	91	0.12	0.35	0.03	91	0.12
p_{12}	0.18	0.18	0.02	93	0.08	0.18	0.02	94	0.08
p_{13}	0.32	0.32	0.03	97	0.12	0.32	0.03	97	0.12
p_{21}	0.37	0.38	0.03	94	0.10	0.38	0.03	95	0.10
p_{22}	0.38	0.39	0.02	96	0.10	0.39	0.02	96	0.10
p_{23}	0.12	0.12	0.01	93	0.05	0.12	0.01	93	0.05
p_{31}	0.33	0.32	0.03	94	0.10	0.33	0.03	93	0.10
p_{32}	0.19	0.19	0.02	95	0.07	0.19	0.02	95	0.07
p_{33}	0.13	0.13	0.01	97	0.05	0.13	0.01	97	0.05
p_{41}	0.39	0.38	0.03	94	0.10	0.39	0.03	94	0.10
p_{42}	0.22	0.22	0.02	95	0.07	0.22	0.02	95	0.07
p_{43}	0.24	0.24	0.02	95	0.07	0.24	0.02	95	0.07
p_{51}	0.39	0.39	0.03	94	0.11	0.39	0.03	94	0.11
p_{52}	0.28	0.27	0.02	92	0.09	0.27	0.02	92	0.09
p_{53}	0.39	0.38	0.03	91	0.11	0.38	0.03	92	0.11

Parameter estimation results of a simulation study with 100 replicates in the setting of $K = 5, T_k = 3$ for $k = 1, \ldots, 5$, $N = 2000$, $\boldsymbol{\beta} = (0.23, 0.21, 0.28, 0.14, 0.14)$, $\boldsymbol{\phi} = (0.76, 0.50, 0.63, 0.62)$, and $\boldsymbol{p} = (0.34, 0.18, 0.32, 0.37, 0.38, 0.12, 0.33, 0.19, 0.13, 0.39, 0.22, 0.24, 0.39, 0.28, 0.39)$. RMSE: root mean square error. CIC%, and CIW represent 95% confidence interval coverage, and mean 95% confidence interval width.

Table 4: Results of Supplementary Simulation 4

		Original				Penalized			
	True	Mean	RMSE	CIC%	CIW	Mean	RMSE	CIC%	CIW
N	3000.00	3005.48	66.26	93	250.52	3003.17	64.71	93	247.36
ϕ_1	0.59	0.59	0.06	92	0.20	0.59	0.05	92	0.20
ϕ_2	0.81	0.80	0.05	96	0.24	0.80	0.05	96	0.23
ϕ_3	0.91	0.91	0.06	91	0.48	0.90	0.05	91	0.28
ϕ_4	0.63	0.63	0.08	95	0.28	0.63	0.07	95	0.27
ϕ_5	0.55	0.55	0.09	95	0.28	0.55	0.08	95	0.27
β_1	0.20	0.20	0.01	95	0.05	0.20	0.01	95	0.05
β_2	0.20	0.20	0.02	97	0.08	0.20	0.02	97	0.08
β_3	0.17	0.17	0.03	96	0.10	0.17	0.02	96	0.10
β_4	0.12	0.12	0.02	98	0.09	0.12	0.02	98	0.09
β_5	0.13	0.14	0.02	96	0.08	0.13	0.02	97	0.08
β_6	0.18	0.18	0.02	93	0.07	0.18	0.02	93	0.07
p_{11}	0.31	0.31	0.02	97	0.10	0.31	0.02	97	0.10
p_{12}	0.26	0.26	0.02	97	0.09	0.26	0.02	97	0.09
p_{13}	0.34	0.34	0.03	97	0.10	0.34	0.03	97	0.10
p_{21}	0.39	0.39	0.03	96	0.13	0.39	0.03	96	0.13
p_{22}	0.13	0.13	0.01	97	0.06	0.13	0.01	97	0.06
p_{31}	0.18	0.18	0.02	97	0.06	0.18	0.02	97	0.06
p_{32}	0.25	0.25	0.02	93	0.08	0.25	0.02	93	0.08
p_{41}	0.20	0.20	0.02	96	0.06	0.20	0.02	96	0.06
p_{42}	0.27	0.27	0.02	94	0.07	0.27	0.02	94	0.07
p_{43}	0.18	0.18	0.01	97	0.05	0.18	0.01	96	0.05
p_{51}	0.16	0.16	0.02	94	0.08	0.16	0.02	93	0.08
p_{52}	0.22	0.22	0.03	96	0.10	0.22	0.03	95	0.10
p_{61}	0.37	0.37	0.04	92	0.13	0.37	0.04	91	0.13
p_{62}	0.27	0.27	0.03	91	0.10	0.27	0.03	91	0.10

Parameter estimation results of a simulation study with 100 replicates in the setting of $K = 6, T_k$ = 3,2,2,3,2,2 for $k = 1, \ldots, 6$, N = 3000, $\boldsymbol{\beta}$ = (0.20, 0.20, 0.17, 0.12, 0.13, 0.18), $\boldsymbol{\phi}$ = (0.59, 0.81, 0.91, 0.63, 0.55), and $\boldsymbol{p}$ = (0.31, 0.26, 0.34, 0.39, 0.13, 0.18, 0.25, 0.20, 0.27, 0.18, 0.16, 0.22, 0.37, 0.27). RMSE: root mean square error. CIC%, and CIW represent 95% confidence interval coverage, and mean 95% confidence interval width.

Table 5: Results of Supplementary Simulation 5

		Original				Penalized			
	True	Mean	RMSE	CIC%	CIW	Mean	RMSE	CIC%	CIW
N	5000.00	4998.40	67.08	98	291.93	4998.90	78.67	95	288.88
ϕ_1	0.90	0.90	0.07	92	0.51	0.88	0.06	92	0.30
ϕ_2	0.85	0.86	0.05	97	0.22	0.85	0.05	96	0.20
ϕ_3	0.80	0.80	0.04	94	0.16	0.80	0.04	97	0.16
ϕ_4	0.75	0.76	0.04	98	0.17	0.75	0.04	96	0.16
β_1	0.10	0.10	0.02	95	0.08	0.10	0.02	95	0.08
β_2	0.15	0.15	0.02	100	0.10	0.15	0.02	97	0.10
β_3	0.20	0.20	0.02	96	0.08	0.20	0.02	97	0.08
β_4	0.25	0.25	0.02	99	0.08	0.25	0.02	95	0.07
β_5	0.30	0.30	0.02	94	0.06	0.30	0.02	95	0.06
p_{11}	0.11	0.11	0.03	93	0.10	0.11	0.02	97	0.10
p_{12}	0.12	0.12	0.03	94	0.11	0.12	0.03	92	0.10
p_{13}	0.13	0.13	0.03	97	0.11	0.13	0.03	97	0.11
p_{21}	0.14	0.14	0.02	95	0.06	0.14	0.01	96	0.06
p_{22}	0.15	0.15	0.02	94	0.06	0.15	0.01	97	0.06
p_{23}	0.16	0.16	0.02	95	0.06	0.16	0.02	95	0.06
p_{31}	0.17	0.17	0.01	93	0.05	0.17	0.01	98	0.05
p_{32}	0.18	0.18	0.01	97	0.05	0.18	0.01	92	0.05
p_{33}	0.19	0.19	0.01	97	0.05	0.19	0.01	94	0.05
p_{41}	0.20	0.20	0.01	95	0.04	0.20	0.01	98	0.04
p_{42}	0.21	0.21	0.01	95	0.04	0.21	0.01	94	0.04
p_{43}	0.22	0.22	0.01	96	0.04	0.22	0.01	97	0.04
p_{51}	0.23	0.23	0.01	94	0.04	0.23	0.01	95	0.04
p_{52}	0.24	0.24	0.01	93	0.05	0.24	0.01	93	0.05
p_{53}	0.25	0.25	0.01	96	0.05	0.25	0.01	92	0.05

Parameter estimation results of a simulation study with 100 replicates in the setting of $K = 5, T_k = 3$ for $k = 1, \ldots, 5, N = 5000, \boldsymbol{\beta} = (0.10, 0.15, 0.20, 0.25, 0.30), \boldsymbol{\phi} = (0.90, 0.85, 0.80, 0.75)$, and $\boldsymbol{p} = (0.11, 0.12, \ldots, 0.25)$. RMSE: root mean square error. CIC%, and CIW represent 95% confidence interval coverage, and mean 95% confidence interval width.

Table 6: Supplementary Model Selection 1

Likelihood	$p(t)\phi(k)$	$p(\cdot)\phi(k)$	$p(t)\phi(\cdot)$	$p(\cdot)\phi(\cdot)$
Original	85	0	15	0
Penalized	83	0	17	0

Summary of a simulation for model selection in the setting of $K = 6, T_k = 2$ for $k = 1, \ldots, 6$, $N = 1000$, $\boldsymbol{\beta} = (0.10, 0.24, 0.11, 0.12, 0.18, 0.25), \boldsymbol{\phi} = (0.87, 0.82, 0.93, 0.54, 0.52)$, and $\boldsymbol{p} = (0.32, 0.27, 0.30, 0.26, 0.22, 0.34, 0.38, 0.18, 0.24, 0.45, 0.19, 0.31)$. Each entry of the table gives the number of cases (out of 100) where the model has the lowest AIC value and is selected as the preferred model.

Table 7: Supplementary Model Selection 2

Likelihood	$p(t)\phi(k)$	$p(\cdot)\phi(k)$	$p(t)\phi(\cdot)$	$p(\cdot)\phi(\cdot)$
Original	96	0	4	0
Penalized	94	0	6	0

Summary of a simulation for model selection in the setting of $K = 6, T_k = 2$ for $k = 1, \ldots, 6$, $N = 2000$, $\boldsymbol{\beta} = (0.10, 0.24, 0.11, 0.12, 0.18, 0.25), \boldsymbol{\phi} = (0.87, 0.82, 0.93, 0.54, 0.52)$, and $\boldsymbol{p} = (0.27, 0.22, 0.25, 0.21, 0.17, 0.29, 0.33, 0.13, 0.19, 0.40, 0.14, 0.26)$. Each entry of the table gives the number of cases (out of 100) where the model has the lowest AIC value and is selected as the preferred model.

Table 8: Supplementary Model Selection 3

Likelihood	$p(t)\phi(k)$	$p(\cdot)\phi(k)$	$p(t)\phi(\cdot)$	$p(\cdot)\phi(\cdot)$
Original	88	0	12	0
Penalized	87	0	13	0

Summary of a simulation for model selection in the setting of $K = 5, T_k = 3$ for $k = 1, \ldots, 5$, $N = 2000$, $\boldsymbol{\beta} = (0.23,\ 0.21,\ 0.28,\ 0.14,\ 0.14)$, $\boldsymbol{\phi} = (0.76,\ 0.50,\ 0.63,\ 0.62)$, and $\boldsymbol{p} = (0.34,\ 0.18,\ 0.32,\ 0.37,\ 0.38,\ 0.12,\ 0.33,\ 0.19,\ 0.13,\ 0.39,\ 0.22,\ 0.24,\ 0.39,\ 0.28,\ 0.39)$. Each entry of the table gives the number of cases (out of 100) where the model has the lowest AIC value and is selected as the preferred model.

Table 9: Supplementary Model Selection 4

Likelihood	$p(t)\phi(k)$	$p(\cdot)\phi(k)$	$p(t)\phi(\cdot)$	$p(\cdot)\phi(\cdot)$
Original	99	0	1	0
Penalized	99	0	1	0

Summary of a simulation for model selection in the setting of $K = 6, T_k =$ 3,2,2,3,2,2 for $k = 1, \ldots, 6$, $N = 3000$, $\boldsymbol{\beta} =$ (0.20, 0.20, 0.17, 0.12, 0.13, 0.18), $\boldsymbol{\phi} =$ (0.59, 0.81, 0.91, 0.63, 0.55), and $\boldsymbol{p} =$ (0.31, 0.26, 0.34, 0.39, 0.13, 0.18, 0.25, 0.20, 0.27, 0.18, 0.16, 0.22, 0.37, 0.27). Each entry of the table gives the number of cases (out of 100) where the model has the lowest AIC value and is selected as the preferred model.

Table 10: Supplementary Model Selection 5

Likelihood	$p(t)\phi(k)$	$p(\cdot)\phi(k)$	$p(t)\phi(\cdot)$	$p(\cdot)\phi(\cdot)$
Original	58	0	42	0
Penalized	46	0	54	0

Summary of a simulation for model selection in the setting of $K = 5, T_k = 3$ for $k = 1, \ldots, 5, N = 5000, \boldsymbol{\beta} = (0.10, 0.15, 0.20, 0.25, 0.30), \boldsymbol{\phi} = (0.90, 0.85, 0.80, 0.75)$, and $\boldsymbol{p} = (0.11, 0.12, \ldots, 0.25)$. Each entry of the table gives the number of cases (out of 100) where the model has the lowest AIC value and is selected as the preferred model.

D Assessment of Initial Parameter Estimates

Table 11: Alternative Initial Values

Parameter	Original	$p_k = 0.10$	$p_k = 0.40$
N	7117.43	8420.12	2708.76
$\widetilde{\phi}_1$	0.14	0.20	0.05
$\widetilde{\phi}_2$	0.12	0.47	0.12
$\widetilde{\phi}_3$	0.13	0.26	0.07
$\widetilde{\phi}_4$	0.27	0.45	0.11
$\widetilde{\phi}_5$	0.67	0.15	0.04
$\widetilde{p}_1$	0.18	0.10	0.40
$\widetilde{p}_2$	0.14	0.10	0.40
$\widetilde{p}_3$	0.38	0.10	0.40
$\widetilde{p}_4$	0.20	0.10	0.40
$\widetilde{p}_5$	0.17	0.10	0.40
$\widetilde{p}_6$	0.02	0.10	0.40
$\widetilde{\beta}_1$	0.33	0.50	0.39
$\widetilde{\beta}_2$	0.07	0.04	0.09
$\widetilde{\beta}_3$	0.01	0.02	0.06
$\widetilde{\beta}_4$	0.20	0.32	0.26
$\widetilde{\beta}_5$	0.11	0.08	0.15

Alternative sets of initial values considered in the assessment of convergence for the selected model of the golden mantella data. The first column presents the initial values computed by applying the methods in Section B of the Supporting Information directly. The second and third columns present values obtained by first setting $p_1 = \ldots = p_6 = 0.10$ (middle) or $p_1 = \ldots = p_6 = 0.40$ (right) and then computing initial values for the remaining parameters as described in Section B of the Supporting Information.

Table 12: Parameter Estimates 2

Parameter	Original	$p_k = 0.10$	$p_k = 0.40$
N	5567(5145,6063)	5472(5028,6002)	5538(5084,6080)
ϕ_1	0.5(0.42,0.58)	0.5(0.42,0.58)	0.54(0.45,0.62)
ϕ_2	0.98(0.78,1)	0.98(0.77,1)	0.98(0.78,1)
ϕ_3	0.66(0.55,0.76)	0.65(0.54,0.75)	0.68(0.56,0.78)
ϕ_4	0.37(0.3,0.45)	0.36(0.29,0.44)	0.38(0.31,0.46)
ϕ_5	0.85(0.21,0.99)	0.73(0.18,0.97)	0.77(0.18,0.98)
β_1	0.44(0.39,0.49)	0.44(0.39,0.5)	0.44(0.39,0.49)
β_2	0.18(0.13,0.24)	0.19(0.14,0.25)	0.19(0.14,0.26)
β_3	0.01(0,0.09)	0.01(0,0.09)	0.01(0,0.09)
β_4	0.13(0.09,0.18)	0.13(0.09,0.18)	0.13(0.09,0.18)
β_5	0.11(0.08,0.13)	0.11(0.09,0.14)	0.11(0.08,0.14)
β_6	0.14(0.08,0.22)	0.12(0.07,0.21)	0.13(0.07,0.22)
N_1	2427(2184,2697)	2427(2184,2696)	2427(2184,2697)
N_2	2232(1891,2635)	2238(1894,2644)	2355(1997,2777)
N_3	2229(1883,2639)	2232(1883,2646)	2350(1987,2779)
N_4	2192(1948,2467)	2171(1928,2444)	2302(2044,2592)
N_5	1403(1196,1646)	1369(1166,1608)	1481(1259,1742)
N_6	1948(1223,3104)	1664(867,3194)	1854(1010,3402)

Point estimates and 95% confidence intervals of the demographic parameters from the selected model fit to the golden mantella data with both penalization and prefiltering starting from different initial parameter estimates. The first column presents the results based on initial parameter estimates derived as described in Section B of the Supporting Information. The second column presents results based on first setting $p_1 = \ldots = p_6 = 0.10$ (middle) or $p_1 = \ldots = p_6 = 0.40$ (right) and then computing initial values for the remaining parameters as described in Section B of the Supporting Information.

Figure 1: Estimated Capture Probabilities 3

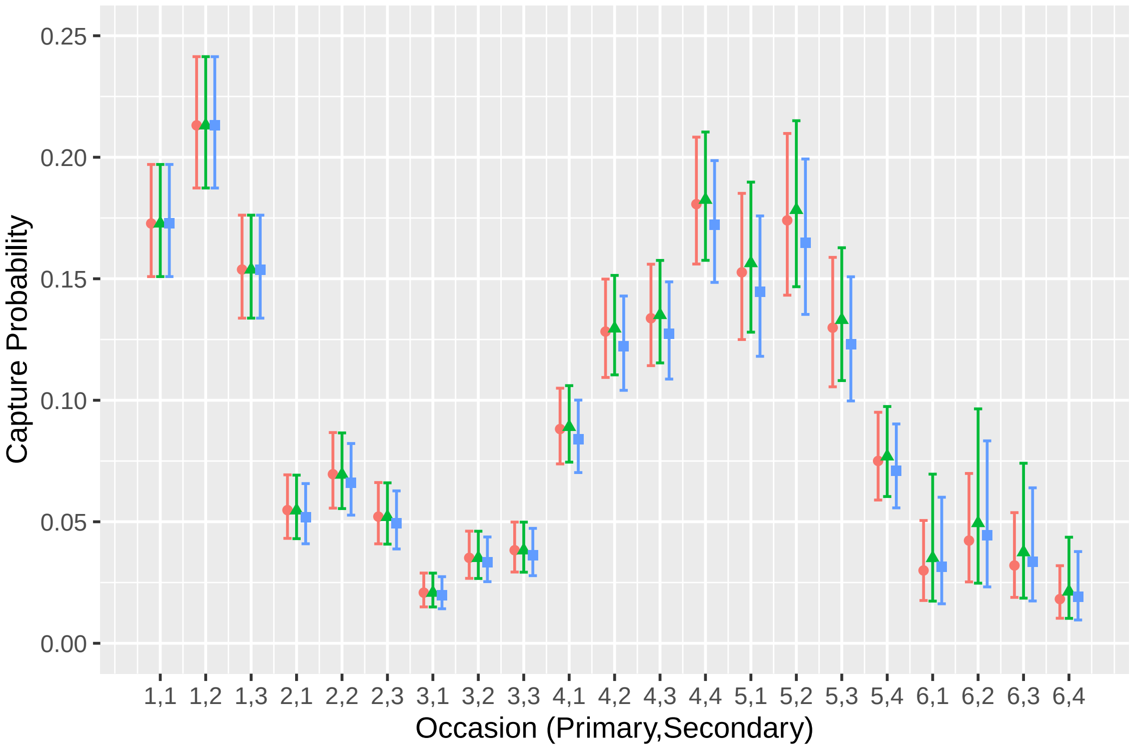

Estimates of the capture probabilities from the selected model using the prefiltered histories with penalization starting from either the initial values computed as described in Section B of the Supporting Information (blue squares) or by first setting $p_1 = \ldots = p_6 = .10$ (green triangles) or $p_1 = \ldots = p_6 = .40$ (red circles) and then computing initial values for the remaining parameters as described in Section B of the Supporting Information. Vertical bars show the extents of the 95% confidence intervals. Points for each version of the model have been offset to avoid overlap. This figure appears in color in the electronic version of this article, and color refers to that version.
